# Optimizing Inpatient Care for Lung Cancer Patients with Immune Checkpoint Inhibitor- Related Pneumonitis Using a Clinical Care Pathway Algorithm

**DOI:** 10.21203/rs.3.rs-4209489/v1

**Published:** 2024-04-12

**Authors:** Norman Brito-Dellan, Maria Cecilia Franco-Vega, Juan Ignacio Ruiz, Maggie Lu, Hadeel Sahar, Pramuditha Rajapakse, Heather Y. Lin, Christine Peterson, Daniel Leal Alviarez, Haider Altay, Sophy Tomy, Joanna-Grace Mayo Manzano

**Affiliations:** The University of Texas MD Anderson Cancer Center; The University of Texas MD Anderson Cancer Center; The University of Texas MD Anderson Cancer Center; The University of Texas MD Anderson Cancer Center; The University of Texas MD Anderson Cancer Center; University of Massachusetts Chan Medical School; The University of Texas MD Anderson Cancer Center; The University of Texas MD Anderson Cancer Center; The University of Texas MD Anderson Cancer Center; The University of Texas MD Anderson Cancer Center; The University of Texas MD Anderson Cancer Center; The University of Texas MD Anderson Cancer Center

**Keywords:** Immune checkpoint inhibitor-related pneumonitis, clinical care pathway algorithm, onco-hospitalist

## Abstract

**Purpose:**

Immune checkpoint inhibitor-related pneumonitis (ICI-P) is a condition associated with high mortality, necessitating prompt recognition and treatment initiation. This study aimed to assess the impact of implementing a clinical care pathway algorithm on reducing the time to treatment for ICI-P.

**Methods:**

Patients with lung cancer and suspected ICI-P were enrolled, and a multi-modal intervention promoting algorithm use was implemented in two phases. Pre- and post-intervention analyses were conducted to evaluate the primary outcome of time from ICI-P diagnosis to treatment initiation.

**Results:**

Of the 82 patients admitted with suspected ICI-P, 73.17% were confirmed to have ICI-P, predominantly associated with non-small cell lung cancer (91.67%) and stage IV disease (95%). Pembrolizumab was the most commonly used immune checkpoint inhibitor (55%). The mean times to treatment were 2.37 days in the pre-intervention phase and, 3.07 days (*p*=0.46), and 1.27 days (*p*=0.40) in the post-intervention phases 1 and 2, respectively. Utilization of the immunotoxicity order set significantly increased from 0% to 27.27% (p = 0.04) after phase 2. While there were no significant changes in ICU admissions or inpatient mortality, outpatient pulmonology follow-ups increased statistically significantly, demonstrating enhanced continuity of care. The overall mortality for patients with ICI-P was 22%, underscoring the urgency of optimizing management strategies. Notably, all patients discharged on high-dose corticosteroids received appropriate gastrointestinal prophylaxis and prophylaxis against Pneumocystis jirovecii pneumonia infections at the end of phase 2.

**Conclusion:**

Implementing a clinical care pathway algorithm for ICI-P management standardizes care practices and enhances patient outcomes, underscoring the importance of structured approaches.

## Introduction

Immune checkpoint inhibitors (ICIs) have revolutionized cancer treatment by activating the immune system against tumors and improving outcomes in various malignancies^1–5^. While offering promising long-term responses, ICIs can also trigger inflammatory effects collectively known as immune-related adverse events (irAEs), which are believed to arise from immunologic enhancement and disruption of normal immune-system homeostasis. These adverse events can be severe and affect any organ system, even resulting in hospitalization or fatality^6^; irAES can occur alone or in combination (multisystem irAEs or overlap syndromes^7^) and can develop at any time after ICI administration^8^.

Managing irAEs involves several key steps including 1) identifying the irAE through a thorough medical history and physical exam; 2) promptly identifying and evaluating competing diagnoses, including disease progression, infections, or comorbidities; 3) grading the irAE on a scale from 1 to 5 (1 = mild, 2 = moderate, 3 = severe, 4 = life-threatening, and 5 = causing death) using the National Cancer Institute’s Common Terminology Criteria for Adverse Events (CTCAE), version 5.0^9^; 4) consulting an organ specialist, if necessary; 5) initiating immunosuppression, usually through the use of corticosteroids; and 6) modifying the administration of the ICI according to the patient’s needs^10^. Early recognition and intervention are crucial for successful irAE management. Delayed diagnosis and treatment may lead to adverse outcomes, even death, underscoring the importance of maintaining a high suspicion index among clinicians^11^.

Pneumonitis, defined as a focal or diffuse inflammation of the lung parenchyma^12^, is a potentially fatal irAE that manifests as interstitial lung disease. Immune checkpoint inhibitor–related pneumonitis (ICI-P) presents in 4 patterns: 1) organizing pneumonia, 2) nonspecific interstitial pneumonia, 3) hypersensitivity pneumonitis, and 4) diffuse alveolar damage; each has distinctive clinical, radiological, and pathological features^13^. The rates of ICI-P vary by the drug class administered and the tumor type. As monotherapies, PD-1, and PD-L1 inhibitors are associated with a higher incidence of any-grade pneumonitis (2.7%−5%) and high-grade pneumonitis (0.8%−2.0%) than CTLA-4 blockers (any-grade pneumonitis, 1.3%; high-grade pneumonitis, 0.3%). Combinations of PD-1 or PDL-1 with a CTLA-4 inhibitor can increase ICI-P rates, which approach 10% in some studies^14^. The mortality rate from ICI-P is around 10%^6^, and patients who develop ICI-P have worse survival outcomes and require more healthcare than those without ICI-P^15^.

Inpatient ICI-P management needs improvement. Specific targets include the time from ICI-P diagnosis to treatment initiation; chemoprophylaxis for the complications of corticosteroid-based immunosuppression (e.g., gastrointestinal [GI] bleeding, opportunistic infections like *Pneumocystis jirovecii* pneumonia [PJP]) for patients on a glucocorticoid dose equivalent to 20 or more mg/day of prednisone for at least 4 weeks; and timely follow up with oncologists and organ-specific specialists (pulmonologists for the purposes of this study).

With the increasing incidence of irAEs requiring hospitalization, oncology-hospitalists (physicians specialized in inpatient cancer care)^16^ are at the forefront of irAE management. Clinical care pathways rooted in evidence-based knowledge enhance teamwork, standardize practices, streamline care processes, and reduce burnout risk in acute hospital settings^17,18^. While professional oncology organizations offer guidelines for irAE management, none provide a comprehensive care pathway from presentation to follow-up after hospitalization^10,14,19,20^. To address this gap, the Onco-Hospital Medicine (OHM) Service at The University of Texas MD Anderson Cancer Center developed a clinical care pathway algorithm for the inpatient management of ICI-P in lung cancer patients requiring hospitalization, mapping key phases and interventions^21^. The algorithm integrates established guidelines with practical experience, providing information on assessing, grading, and managing ICI-P^22^. It also includes a process for triaging patients with severe acute respiratory syndrome coronavirus 2 (SARS-CoV-2) infection, as the algorithm was developed during the global coronavirus disease 19 (COVID-19) pandemic^23^. The objectives of this clinical care pathway algorithm were to increase the awareness and recognition of ICI-P, facilitate timely diagnosis and treatment, activate a multidisciplinary team for the care of patients with ICI-P, and ensure adequate follow-up after hospital discharge, ultimately leading to better patient outcomes and reduced variations in patient care^24^.

In parallel with the development of the algorithm, the Institutional-led Toxicity Working Group created an inpatient immune-mediated toxicity work-up (immunotoxicity) order set with clinical orders standardizing and expediting the work-up and diagnosis of irAEs, including ICI-P. This order set was integrated into the patients’ electronic health records.

The study aimed to improve the care and outcomes for lung cancer patients suspected of having ICI-P by implementing a clinical care pathway algorithm into daily hospital practice and by developing and disseminating educational materials to encourage clinical staff to use the algorithm.

## METHODS

We conducted a retrospective cohort study of patients with lung cancer who were admitted to the OHM service at MD Anderson Cancer Center with suspicion of ICI-P from January 1, 2020, to December 31, 2022. Patients were included in the study if they 1) had at least 1 diagnosis code for neoplasm of the lung/bronchus or bronchial tree/trachea per the International Classification of Diseases, version 10 (ICD-10)^25^; 2) had received at least 1 ICI (pembrolizumab, nivolumab, ipilimumab, durvalumab, atezolizumab; and 3) were admitted to or discharged from the OHM service during the study period.

The patients’ electronic health records were used to obtain information regarding their demographics and treatments. Patients were classified as having suspected ICI-P if healthcare providers had included ICI-P as part of the differential diagnosis for the patients’ clinical presentations. ICI-P was evaluated further through diagnostic testing and/or consultation with a pulmonologist. Patients were classified as having confirmed ICI-P if there was a consensus regarding the diagnosis at the end of the hospitalization period among the patients’ healthcare providers, including oncology-hospitalists, oncologists, and pulmonologists, that the patient’s clinical presentation was ICI-P or if ICI-P therapy was initiated during hospitalization. Since ICI-P is a diagnosis of exclusion, we excluded from the study any patient with a confirmed or suspected competing diagnosis, including those with an active pulmonary infection like COVID-19, lung cancer progression, radiation-induced pneumonitis, or pneumonitis associated with another therapeutic agent such as a tyrosine-kinase inhibitor.

**Figure 1:**
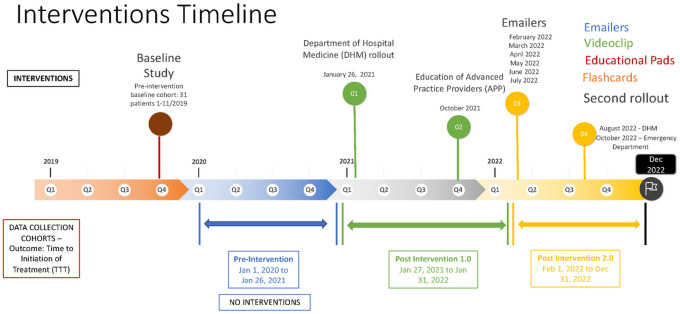
Timeline of interventions

**Figure 2:**
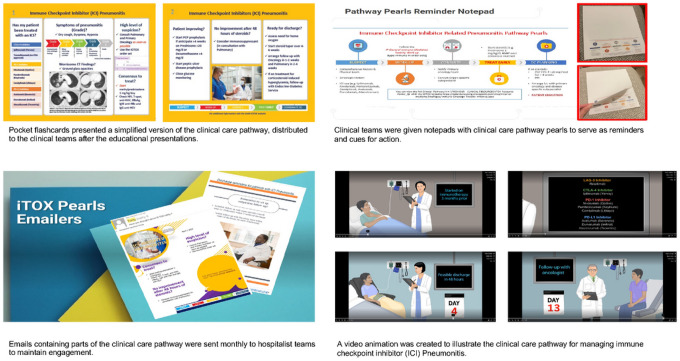
Multimodal interventions

We developed a multimodal intervention to promote the use of the clinical care pathway algorithm in the OHM service. Interventions were rolled out in 2 phases: phase 1 included educational sessions, while phase 2 included the distribution of flashcards and notepads that contained information on the clinical presentation of ICI-P and the clinical care pathway algorithm. Additionally, we sent out monthly reminder emails and developed and presented a videoclip animation of the clinical care pathway algorithm (Figures 1 and 2). The primary outcome of our study was the time to the first ICI-P treatment, i.e., the time to treatment before and after implementation of the clinical care pathway. Secondary outcomes included the ICU admission rate, inpatient mortality rate, length of stay, 30-day unplanned readmission rate, use of the immunotoxicity order set, frequency of pulmonology and oncology consultations, time to the first pulmonology and oncology consultations, use of GI and PJP prophylaxis for patients discharged on high doses of corticosteroids (a dose of prednisone or its equivalent of ≥20 mg/day), and time to the first post-discharge follow-up with the pulmonary and oncology services.

We used descriptive statistics [frequency distribution, mean (± s.d.), and median (range)] to summarize patients’ characteristics. We used the Kruskal-Wallis test to compare the time to treatment between the pre-intervention and post-intervention phases. P-values less than 0.05 were considered statistically significant. All analyses were conducted using SAS (version 9.4, Cary, NC) software. The study was approved by the Quality Improvement Approval Board at MD Anderson.

## RESULTS

Of the 82 patients admitted with a suspicion of ICI-P, 60 (73.17%) had confirmed ICI-P, 64 (78.05%) received an ICI, and the immunotoxicity order set was used in 10 (12.20%).

Of those with confirmed ICI-P, 19 (31.67%) patients were included in the pre-intervention group (from January 1, 2020, to January 26, 2021), 30 (50.00%) were included in post-intervention phase 1 (from January 27, 2021, to January 31, 2022), and 11 (18.33%) were included in post-intervention phase 2 (from February 1, 2022, to December 31, 2022). Fifty-five (91.67%) of the patients in our cohort of patients with confirmed ICI-P had NSCLC, and 57 (95.00%) patients had stage IV disease. Thirty-ve (58.33%) were men, and 47 (78.33%) were White. The mean age of the patients at admission was 66.55 years (range, 38.03–84.9 years). Pembrolizumab, a PD-1–receptor blocker, was the most-used ICI (33 [55.00%] patients), followed by the combination of ipilimumab + nivolumab (9 [15.00%] patients). Forty-eight (80.00%) patients were on active immunotherapy at the time of admission. Twenty-five (41.67%) patients had received 3 doses or less of an ICI before admission. Fifty-five (91.67%) patients presented with a respiratory complaint (e.g., dyspnea) on admission, and 13 (21.67%) had a concurrent irAE in addition to ICI-P. All patients had severe ICI-P (grade 33 per the CTCAE, version 5.0). Fifty-nine (98.33%) patients received corticosteroids for the treatment of ICI-P, and 10 (16.67%) also received infliximab for steroid-refractory ICI-P. A pulmonology consultation was requested for 59 (98.33%) patients, and the mean time between the ICI-P diagnosis and the consultation was 2.53 days (range, 0.00–16.0 days) (Table 1).

The mean time to treatment was 2.37 days (range, 0–12 days) in the pre-intervention phase, 3.07 days (range, 0–17 days) in post-intervention phase 1 (p = 0.46 for the pre-intervention phase versus post-intervention phase 1), and 1.27 days (range, 0–6 days) in post-intervention phase 2 (p = 0.40 for the pre-intervention phase versus post-intervention phase 2). Use of the immunotoxicity order set increased from 0% during the pre-intervention phase to 20% after phase 1 (*p* = 0.07) and 27.27% after phase 2 (*p* = 0.04). The percentage of patients discharged on high-dose steroids who received prescriptions for PJP prophylaxis increased from 71.43% in the pre-intervention phase to 95.24% in post-intervention phase 1 (*p* = 0.13) and 100% in post-intervention phase 2 (*p* = 0.13). ICU stays were needed in 42.11% of the patients in the pre-intervention phase, 26.67% of those in post-intervention phase 1 (*p* = 0.35), and 27.27% of those in post-intervention phase 2 (*p* = 0.47). The inpatient mortality rate was 26.32% in the pre-intervention phase, 16.67% in post-intervention phase 1 (*p* = 0.48), and 18.18% in post-intervention phase 2 (*p* = 1.00) (Table 2). There were no statistically significant changes in the overall ICU admission or inpatient mortality rates from the pre-intervention phase to the post-intervention phases 1 and 2 (*p* = 0.5 and *p* = 0.6, respectively).

Of the 48 patients discharged alive (Table 3), 41 (85.42%) were on a glucocorticoid dose equivalent to 3 20 mg/day of prednisone; all of these patients were also on GI prophylaxis with a proton-pump inhibitor or a histamine type-2-receptor antagonist, and all were prescribed PJP prophylaxis. Outpatient follow-up with an oncologist was documented in 35 (72.92%) patients, and the median time to first oncology follow-up was 20.0 days. Outpatient follow-up with a pulmonologist increased significantly from 23.1% in the pre-intervention phase to 64% in post-intervention phase 1 (p = 0.0382) and 100% in post-intervention phase 2 (*p* = 0.0031), with an overall *p*-value of 0.0030. The median time to first follow-up with a pulmonologist was 18.5 days.

## DISCUSSION

To our knowledge, this is the first study to evaluate the effectiveness of a clinical care pathway for managing ICI-P in hospitalized patients with lung cancer. The study shows a reduction in the time to initiate ICI-P treatment in this patient population. Additionally, we have increased the usage of the immunotoxicity order set in the electronic health records. Our comprehensive, multimodal intervention played a vital role in encouraging healthcare providers to use the order set. Although the observed change did not reach statistical significance, we believe this approach is a pioneering and unique effort in the field.

In cases where grade 3 or 4 pneumonitis leads to hypoxia or respiratory compromise, hospitalization is required as it can be life-threatening^26–28^. Guidelines for diagnosing and managing irAEs recommend multidisciplinary consultation, high doses of oral or intravenous corticosteroids, and discontinuation of ICI therapy^29–31^.

In our study, 91.67% of confirmed ICI-P patients had NSCLC, and 95% had stage IV. Pembrolizumab was the most common ICI (55%). Glucocorticoids were frequently used (98.33%), while second-line immunosuppressants were rare. This could be due to the low incidence of steroid-refractory ICI-P or hesitancy to initiate advanced immunosuppression without a clearly preferred approach to immunosuppressive therapy. Steroid-refractory ICI-P, an often-fatal clinical phenomenon with poorly understood incidence^32,33^, was identified in 10 (16.67%) patients in our cohort, necessitating escalation to infliximab, a tumor necrosis factor-alpha inhibitor that reduces inflammation and alters the immune response.

A systematic review of 159 studies involving 33,253 patients showed that using glucocorticoids increased the risk of GI bleeding and perforation^34^. Consequently, best-practice guidelines recommend acid suppression for patients at risk of GI bleeding^35^. Within our patient cohort, those discharged while receiving a glucocorticoid dose equal to or greater than 20 mg/day of prednisone were given GI prophylaxis in either a proton-pump inhibitor or a histamine type-2 receptor antagonist. Similarly, all these patients received PJP prophylaxis. Thus, our study’s interventions helped ensure compliance with the recommended best practices to prevent GI complications and opportunistic infections while on glucocorticoids^36^.

We observed no significant changes in ICU admissions or inpatient mortality from the pre-intervention phase to the post-intervention phases 1 and 2 (p = 0.5 and p = 0.6, respectively). The overall mortality rate for patients with ICI-P was 22%, which is higher than the typically reported mortality rate of approximately 10% in the literature^37^. However, our findings are consistent with those of a real-world cohort investigation involving 315 patients with lung cancer who were treated with ICIs in 6 healthcare centers (1 academic center, 1 community referral center, and 4 community centers) within the University of North Carolina network. This study reported an ICI-P incidence rate of 9.5%, with 60% of patients requiring hospitalization for ICI-P management. The risk of mortality within this patient subset was 32%^38^. Therefore, our findings and those of the aforementioned study suggest that ICI-P is more common and severe than previously reported, and it carries an unexpectedly high mortality rate.

Our study’s interventions resulted in more timely follow-up appointments with the oncology and pulmonology services.

It is worth noting that the project and data collection took place during the peak of the COVID-19 pandemic, which posed significant challenges. Clinical presentation and radiological ndings of ICI-P and SARS-CoV-2 can be quite similar; patients with respiratory symptoms needed to be isolated until their SARS-CoV-2 tests were available, causing delays in ICI-P diagnosis and treatment. Moreover, the widespread prevalence of COVID-19 pneumonia created a diagnostic bias, as it was the leading differential diagnosis in most patients with respiratory symptoms.

The study had a limitation in that it relied on billing codes and other coded data to identify ICI-P. This is because there are no specific ICD-10 codes available for the disease. To identify potential cases of ICI-P for the incidence analysis, broad codes were used intentionally. This was because clinicians often use various codes when faced with an uncertain diagnosis of ICI-P.

Bronchoscopy, which is recommended in irAE management guidelines, is infrequently used in severe ICI-P cases because patients may be clinically unstable and unable to undergo an invasive procedure under anesthesia. Unfortunately, none of the patients in our cohort could undergo diagnostic bronchoscopy due to their clinical instability. The primary value of invasive bronchoscopy is identifying alternative etiologies for the patient’s symptoms (e.g., disease progression, infectious pneumonia)^39^. Noninvasive alternatives like diagnostic biomarkers for ICI-P may be preferable but remain elusive. Future studies of ICI-P should focus on describing its clinical features more accurately and optimizing its diagnostic algorithms, given the current lack of a gold standard for diagnosis.

## CONCLUSION

This study provides valuable insights into the management and outcomes of patients with lung cancer who exhibit symptoms of ICI-P (Immune Checkpoint Inhibitor-Related Pneumonitis). It emphasizes the critical role of onco-hospitalists in managing severe cases of ICI-P that require hospitalization. By implementing a clinical care pathway algorithm based on evidence, the variability in the time taken to administer treatment was reduced, and there was a significant increase in the use of the immunotoxicity order set. Consequently, the implementation led to the standardization of clinical care. Importantly, the study underscores the feasibility of implementing best practices in patient care even outside the confines of comprehensive cancer centers, making these practices relevant and applicable to nononcologists and healthcare practitioners in diverse clinical contexts.

At our institution, continual educational efforts to bolster adherence to the established care pathway algorithm and enhance patient outcomes will be imperative.

## Figures and Tables

**Table 1.** Patients’ characteristics.

Characteristic	Patients with suspected ICI-P n = 82	Patients with confirmed ICI-P n = 60
Age at admission in years, mean (SD)	66.36 (11.28)	66.55 (12.14)
Intervention phase, n (%)		
Pre-intervention	22 (26.83)	19 (31.67)
Post-intervention 1	36 (43.90)	30 (50.00)
Post-intervention 2	24 (29.27)	11 (18.33)
Consensus on ICI-P diagnosis, n (%)	60 (73.17)	N/A
Sex, n (%)		
Men	48 (58.54)	35 (58.33)
Women	34 (41.46)	25 (41.67)
Race, n (%)		
American Indian*	1 (1.22)	1 (1.67)
Asian	5 (6.10)	5 (8.33)
Black^+^	5 (6.10)	3 (5.00)
White	66 (80.49)	47 (78.33)
Other^	5 (6.10)	4 (6.67)
Ethnicity, n (%)		
Hispanic or Latino	10 (12.20)	7 (11.67)
Not Hispanic or Latino	71 (86.59)	53 (88.33)
Declined to answer	1 (1.22)	-
Lung cancer type, n (%)		
NSCLS	73 (89.02)	55 (91.67)
SCLC	9 (10.98)	5 (8.33)
Cancer stage at admission, n (%)		
Stage III	10 (12.20)	3 (5.00)
Stage IV	72 (87.80)	57 (95.00)
Active treatment with ICI, n (%)	64 (78.05)	48 (80.00)
Type of ICI, n (%)		
Atezolizumab	11 (13.41)	7 (11.67)
Durvalumab	11 (13.41)	7 (11.67)
Durvalumab + tremelimumab	2 (2.44)	1 (1.67)
Ipilimumab + nivolumab	15 (18.29)	9 (15.00)
Nivolumab	4 (4.88)	3 (5.00)
Pembrolizumab	39 (47.56)	33 (55.00)
Number of ICI doses received before admission, n (%)		
≤ 3	36 (43.90)	25 (41.67)
4–6	21 (25.61)	15 (25.00)
7–9	8 (9.76)	6 (10.00)
≥ 10	17 (20.73)	14 (23.33)
First line of ICI therapy, n (%)	66 (80.49)	45 (75.00)
Respiratory complaint on admission, n (%)	71 (86.59)	55 (91.67)
Use of immunotoxicity order set, n (%)	10 (12.20)	9 (15.00)

ICI, immune checkpoint inhibitor; ICI-P, immune checkpoint inhibitor–related pneumonitis; ICU, intensive care unit; N/A, not applicable; NSCLS, non-small cell lung cancer; SCLC, small cell lung cancer; SD, standard deviation.

* Including Alaska Natives.

+ Including African Americans.

^ Including self-reported mixed races and other races not otherwise specified

Of the 60 patients with confirmed ICI-P, 59 (98.33%) patients received corticosteroids for the treatment of ICI-P, and 10 (16.67%) also received infliximab for steroid-refractory ICI-P. Pulmonology was consulted in 59 (98.33%) patients, and the mean time between the ICI-P diagnosis and the consultation was 2.53 days (range, 0.00–16.0 days). Oncology was consulted in 37 (61.67%) patients.

**Table 2.** Patient characteristics by intervention phase among patients with confirmed ICI-P (N= 60).

Characteristic	Pre-intervention phase	Post-intervention phase 1	p-value (post-intervention phase 1 versus pre-intervention phase)	Post-intervention phase 2	*p*-value (post-intervention phase 2 versus pre-intervention phase)
Time to treatment of ICI-P in days, mean (min-max)	2.37 (0–12)	3.07 (0–17)	0.46	1.27 (0–6)	0.40
Use of immunotoxicity order set (N = 60), n (%)	0/19 (0.00)	6/30 (20.00)	0.07	3/11 (27.27)	0.04
ICU stay (N = 60), n (%)	8/19 (42.11)	8/30 (26.67)	0.35	3/11 (27.27)	0.47
Inpatient mortality (N = 60), n (%)	5/19 (26.32)	5/30 (16.67)	0.48	2/11 (18.18)	1.00
Length of hospital stay in days, mean (min-max)	17.68 (2–41)	12.60 (3–28)	0.0418	13.82 (3–38)	0.2037
Pulmonology consultation, n (%)	18/19 (94.74)	30/30 (100)	0.3878	11/11 (100)	1.00
Primary Oncology consultation, n (%)	10/14 (71.43)	12/25 (48.00)	0.1935	3/9 (33.33)	0.1023
Discharged with PPI/H2 blockers if on steroids (N = 45*), n (%)	12/12 (100)	19/23 (82.61)	0.29	9/9 (100)	N/A
Discharged with PJP prophylaxis if on steroids (≥ 20 mg/day prednisone) (N = 46*), n (%)	10/14 (71.43)	20/21 (95.24)	0.13	9/9 (100)	0.13
Readmission (N = 12^+^), n (%)	5/14 (35.71)	5/23 (21.74)	0.4537	2/8 (25.00)	1.000
30-day mortality (N = 48), n (%)	3/14 (21.43)	7/25 (28.00)	0.7212	1/9 (11.11)	1.0000
Follow-up pulmonology appointment arranged if treated for ICI-P n (%)	7/14 (50.00)	19/25 (76.00)	0.1574	8/9 (88.9)	0.0858
Patient received outpatient pulmonology follow-up, n (%)	3/13 (23.10)	16/25 (64.00)	0.0382	6/6 (100)	0.0031
Time to first pulmonology follow-up in days, mean (min-max)	43.44 (4–111)	24.16 (1–99)	0.3757	32.75 (5–110)	0.8097
Time to first oncology follow-up in days, mean (min-max)	43.44 (4–111)	25.00 (1–99)	0.3958	32.75 (5–110)	0.8097

H2, histamine type-2 receptor; ICI-P, immune checkpoint inhibitor–related pneumonitis; ICU, intensive care unit; max, maximum; min, minimum; PJP, Pneumocystis jirovecii pneumonia; PPI, proton-pump inhibitor.

* One patient in the pre-intervention phase had missing data on PPI use, and 2 patients (1 in the pre-intervention phase and 1 in the post-intervention phase) had missing data on prednisone use.

+ Of the 48 patients alive at discharge, 12 (25.00%) patients were readmitted within 30 days (all cause-readmissions). No data were available for 3 (6.25%) of the 48 patients.

**Table 3.** Inpatient mortality and discharge dispositions for patients with confirmed ICI-P (N = 60).

Patient outcome	n (%)
Died as inpatient	12 (20.00)
Discharged	48 (80.00)
To home without home health or physical therapy services	25 (52.08)
To home with home health or physical therapy services	9 (18.75)
To skilled nursing facility	7 (14.58)
To home with hospice services	4 (8.33)
To acute care hospital	1 (2.08)
To another health care institution	1 (2.08)
To a rehabilitation facility	1 (2.08)

ICI-P, immune checkpoint inhibitor–related pneumonitis.
